# IFNα Inducible Models of Murine SLE

**DOI:** 10.3389/fimmu.2013.00306

**Published:** 2013-10-02

**Authors:** Zheng Liu, Anne Davidson

**Affiliations:** ^1^Center for Autoimmune and Musculoskeletal Diseases, The Feinstein Institute for Medical Research, Manhasset, New York, NY, USA

**Keywords:** interferon α, lupus, nephritis, mouse model, inflammation

## Abstract

The role of type I interferons (IFNs) in SLE pathogenesis has been a subject of intense investigation in the last decade. The strong link between type I IFNs and SLE was initially provided by *ex vivo* studies showing that exposure of peripheral blood mononuclear cells to immune complexes from SLE patients elicits a signature of IFN inducible genes and was then further highlighted by human genetic studies. The mechanisms by which type I IFNs, especially IFN alpha (IFNα), modulate the immune system and exacerbate SLE have been largely elucidated through studies in mouse lupus models. In this review, we discuss the characteristics of several such models in which disease is accelerated by ectopically expressed IFNα. We also summarize several studies which tested therapeutic interventions in these models and discuss the advantages and disadvantages of using IFNα accelerated models to study experimental treatments for lupus.

## Introduction

In the last decade, type I interferons (IFNs) have received particular attention for their role in the pathogenesis of systemic lupus erythematosus (SLE). The induction of anti-dsDNA antibodies and development of lupus-like symptoms in a small number of IFN treated patients with cancer or infectious diseases suggested a causal link between this cytokine and SLE (1). The discovery of the “IFNα signature,” which refers to the augmented expression of a group of IFNα induced genes, in peripheral blood mononuclear cells (PBMCs) from active lupus patients further highlighted the essential role of type I IFNs in the disease (2, 3). The IFN signature is induced in healthy PBMCs by SLE plasma containing nucleic acid associated immune complexes and this induction is inhibited by anti-IFNα antibody (4). Furthermore, polymorphisms in several genetic loci that are involved in the toll-like receptor (TLR)/IFN signaling pathway are associated with SLE risk (5, 6). These reports establish the important role of IFNα in the pathogenesis of SLE and are the basis for the development of drugs that target Type I IFNs or their receptor.

Type I IFNs are produced by several different cell types and are of major importance in anti-viral defense. In conventional dendritic cells, Type I IFN production is triggered by a several mechanisms including activation of endosomal TLR3 and binding to cytosolic nucleic acid receptors [reviewed (7) – Table 1]. By contrast, plasmacytoid dendritic cells (pDCs) are a major source of Type I IFN in SLE. Opsonized apoptotic material or circulating immune complexes of self-nucleic acids and autoantibodies are taken up by pDCS through the Fcγ receptor (8) and their nucleic acid components can then traffic to the endosome where they interact with TLR7 or TLR9. The adaptor molecule MyD88 is then recruited and this results in phosphorylation of IRAK1 and activation of the transcription factor IRF7 that induces IFN production. pDCs rapidly produce large amounts of IFNs owing to their constitutive expression of IRF7 (1, 9–11). Recent studies have shown that cytosolic DExD/H-Box helicases can sense cytoplasmic DNA (12) and initiate type I IFN production in human pDCs through the IRF7 pathway (13). The interaction between DNA and TLR9 is facilitated by a nuclear DNA binding protein HMGB1 (14) which is a component of neutrophil derived neutrophil extracellular traps (NETs) released from dying neutrophils in SLE (15). Other components of NETs protect nucleic acids from degradation and enhance their ability to form stable immune complexes with SLE related autoantibodies (16).

**Table 1:** **Induction and pro-inflammatory effects of Type I interferons**.

Cell type	Effect	Mechanisms	Reference
B cells	Induction of autoantibodies	Enhancement of response to TLR activation	(18, 35)
		Upregulation of MHCII, CD86, CD69	
	Induction of germinal centers	Increased class switch to pathogenic isotypes IgG2a and IgG3	
		Dysregulation of CD62L expression with increased shuttling of antigen from the MZ to the follicles	
	Induction of plasma cells	Enhanced crosstalk with IL-6 signaling	
		Induction of miR-15a and repression of PAX5	
		Increased expression of BLIMP and XBP	
	Induction of short-lived plasma cells	Decreased bone marrow expression of CXCL12 and VCAM-1	(34)
Conventional dendritic cells	Release of Type I IFN in response to TLR3 activation	TRAF3 mediated recruitment of TBK1, IKKϵ, and IRF3, leading to IRF3 phosphorylation	(7, 17)
	Release of Type I IFNS through receptors for cytosolic nucleic acids	RIG-I mediated recruitment of the MAVS adapter and mitochondrial membrane assembly of a TRAF3, TBK1, and IKKϵ signalosome leading to IRF3 phosphorylation	
		IFI16 mediated induction of a STING, TBK, IRF3-dependent pathway	
	Priming for antigen presentation	Upregulation of MHC and costimulatory molecules	
		Increased expression of CCR7	
	Release of cytokines including BAFF	TRAF6 mediated NFκB activation	
Plasmacytoid dendritic cells	Rapid release of high concentrations of Type IFN in response to immune complexes	High levels of endosomal TLR7, 8, and 9 and activation of the MyD88 adaptor	
		Constitutive expression of IRF7	
		Formation of late endosomes	
T cells	CD4 T cell stimulation	Increased IFNγ production	(7, 24)
		Enhanced survival	
	Priming for induction of CD8 killer cells	Increased cross-presentation	
		Increased gene transcription	
		Enhanced responsiveness to IL-2 and IL-15	
	Decreased Treg function	Downregulation of intracellular cAMP	(62)

Type I IFNs can be induced in conventional DCS and macrophages following activation of TNFR1 and LTβR receptors. In addition, intracytoplasmic nucleic acids may trigger cytoplasmic receptors and activate a mitochondrial membrane pathway culminating in phosphorylation of IRF7 and Type I IFN production [reviewed (7)]. A complete description of the molecular pathways involved in type I IFN production in SLE is beyond the scope of this article but is the subject of several recent reviews (7, 17).

Type I IFNs have profound effects on the innate and adaptive immune systems [reviewed (7, 17, 18) – Table 1]. Serum from SLE patients induces monocytes from healthy donors to acquire a DC-like phenotype and become potent activators of T cells (19) in an IFNα dependent manner. Furthermore, IFNα acts on conventional DCs to enhance their production of an important B cell survival factor, B cell activating factor (BAFF) (20, 21). IFNα upregulates TLR7 expression on B cells, which in turn mediates increased expression of TACI, a receptor for BAFF (22). Its dual role in promoting BAFF production of DCs and enhancing the responsiveness of B cells to BAFF makes IFNα an important modulator of the fate of autoreactive B cells. Furthermore, IFNα drives B cell differentiation into CD138+ plasmablasts; terminal differentiation into Ig-secreting plasma cells is mediated by IL-6, another cytokine produced by activated pDCs (23). Finally, type I IFNs stimulate CD4 T cells to enhance antigen-specific B cell responses and prevent activated T cell death in mice (24, 25). These immunological features of type IFNs may all contribute to the pathogenesis of SLE.

Direct evidence for the essential role of IFNs in SLE was achieved through studies using lupus-prone mice that are genetically deprived of type I IFN signaling or treated with exogenous type I IFNs. *Ifnar1* gene deficiency largely protects lupus-prone mice from disease onset or attenuates disease severity (26–29). Conversely, transient overexpression of exogenous IFNα accelerates disease progression in all lupus-prone mice tested to date. This makes these models not only useful tools to understand the role of IFNs in SLE, but also useful platforms to test potential therapies for SLE.

## IFNα Accelerated Lupus Mouse Models

### NZB/W F1 mice

New Zealand black/New Zealand white (NZB/W) F1 mice are a widely used animal model for lupus; they mimic human lupus in several aspects including gender specificity, the appearance of circulating anti-dsDNA antibodies, renal deposition of immune complexes and the development of fatal glomerulonephritis. They do not develop skin disease or hematologic manifestations and thus have been used primarily to study SLE nephritis. NZB/W F1 mice develop proteinuria at a median age of 37 weeks and die by the age of 1 year (30, 31). Although NZB/W F1 mice do not develop detectable levels of circulating IFNα (20), the IFN signature can be detected in splenic cells of pre-autoimmune NZB/W F1 mice (32). The disease-initiating activities of IFNα in NZB/W F1 mice were suggested by a report that treatment with poly IC, a TLR3 agonist, accelerates the disease in these mice (33). More recently, a single injection of an adenovirus expressing IFNα (Ad-IFNα) has been shown to accelerate the production of circulating anti-dsDNA antibodies, renal deposition of immune complexes, onset of proteinuria, and death in NZB/W mice in a dose dependent manner (20, 34). The accelerated clinical manifestations are associated with a vastly enhanced germinal center reaction, increased serum levels of pro-inflammatory cytokines, and the induction of T cell expression of IL-21 (34). This pro-inflammatory environment is associated with expanded B cells, CD4 T cells, and DCs (34) and loss of B10 cells (35). Furthermore, IFNα virus injection induces elevated serum levels of BAFF and increased TLR7 expression on splenic B cells (20–22, 34). Interestingly, although NZB/W F1 mice normally possess a proportion of long-lived autoreactive plasma cells in the spleen and BM, treatment with Ad-IFNα skews the differentiation of autoreactive B cells almost completely toward short-lived plasma cells [(34, 36) reviewed in (37)]. This appears to be due to a decrease in bone marrow expression of CXCL12 and VCAM-1, both of which are components of the bone marrow plasma cell niche (34). Finally, in contrast to conventional mice, Ad-IFNα treated NZB/W F1 mice have far less renal interstitial leukocyte infiltration. This is due to reduced renal expression of pro-inflammatory chemokines such as CXCL13 and intrinsic defects of leukocyte migration toward these chemokines (38).

Most of these features have also been reported in Ad-IFNα treated New Zealand Mixed 2328 mice (39). However, despite a large increase in T cell numbers, these mice do not develop a preferential expansion of memory T cells following IFN treatment or substantial glomerular macrophage infiltration as they age, suggesting these two features may not be driven by type I IFNs. In addition to the immune effects of Type I IFNs in this model, administration of Ad-IFNα has a detrimental effect on the vasculature, causing impairment of endothelium-dependent vasorelaxation, a decrease in maturation of endothelial progenitor cells into mature endothelial cells, increased platelet activation, and accelerated thrombus formation, suggesting a potential role for IFN in the accelerated atherosclerosis associated with SLE (40).

Studies using cell depletion or mice with genetic deficiencies have shown that disease acceleration by IFN is dependent on T cells (NZB/W mice) (34), B cells (NZM2328 mice) and BAFF (NZM2328 mice) (39).

### NZW/BXSB mice

Male NZW/BXSB mice carry two active copies of the TLR7 gene. They develop anti-RNA and anti-phospholipid autoantibodies, severe inflammatory nephritis and anti-phospholipid syndrome with thrombocytopenia, myocardial infarcts, and cardiomyopathy (41, 42). The survival of these mice is prolonged by prophylactic treatment with anti-IFNAR antibody, suggesting the disease process is driven by IFNα (43). In contrast, female mice with a single active copy of TLR7 develop late onset nephritis, but not anti-phospholipid syndrome (42, 44). Administration of Ad-IFNα induced high titers of circulating anti-phospholipid, anti-Sm/RNP, and anti-DNA autoantibodies and markedly accelerated nephritis and death, but not anti-phospholipid syndrome in female NZW/BXSB mice (44). These IFNα induced effects were accompanied by a striking increase in activated B and T cells in the spleen. Using female NZW/BXSB mice bearing the site-directed anti-cardiolipin/DNA autoantibody V_H_ transgene 3H9, IFNα has been shown to relax the stringency for selection against autoreactivity of the antigen selected B cell repertoire (45).

### B6.Sle123

B6.Sle123 mice, that possess three SLE susceptibility loci, spontaneously develop highly penetrant severe systemic autoimmunity and fatal glomerulonephritis beginning at 6 months of age. Young pre-autoimmune mice that were treated with IFNα quickly developed renal immune complex deposition and nephritis, accompanied by increased serum levels of pro-inflammatory cytokines such as TNFα and IL-6, activation of DCs, B cells, and T cells, as well as an enhanced germinal center response (46). As in the strains discussed above, renal leukocyte infiltration was not affected by IFNα treatment.

Collectively, these studies show that excess IFNα accelerates progression of glomerulonephritis in most lupus models. The acceleration and severity of the disease is dose dependent, allowing researchers to control of the duration of their study. IFNα induces a T dependent and enhanced germinal center response and exhibits characteristics of the disease in the conventional strain. For instance, IFNα induces anti-dsDNA antibodies in NZB/W F1 mice and anti-RNA antibodies in BXSB mice, these being the predominant specificities in the respective strains (20, 34, 44). Of interest is the skewing of the antibody response from long-lived to short-lived plasma cells in the NZB/W model, a feature associated with alterations of the bone marrow environment. However, IFN acceleration is associated with less renal inflammatory cell infiltration compared to its spontaneous counterpart. This is probably due to the short disease course which does not allow these features to develop to the same extent as in the conventional mice. It is also important to note that other major manifestations of human SLE including skin, hematologic, and neurologic disease cannot be addressed using these models.

## IFNλ Acceleration of SLE

IFNλ is a family of Type III IFNs that mediate their biologic activities through a receptor that is expressed predominantly on epithelial cells and induce a similar pattern of gene expression as Type I IFNs (47). Treatment of NZB/W mice with a continuous infusion of IFNλ did not exacerbate disease, however the addition of IFNλ to a low dose of IFNα modestly accelerated proteinuria onset (35).

## Association of Type I IFNs with Alteration in miRNAs

Type I IFN production can be regulated by miRNAs. For example, underexpression of miR-146a was found to correlate with disease activity and with an IFN signature. miR-146a targets multiple components of the IFN signaling pathway such that its deficiency results in overexpression of IFN inducible genes. Importantly, administration of TLR agonists or of Type I IFN induced expression of miR-146a indicating a physiologic feedback loop that may be dysregulated in SLE (48). Delivery of miR-146a to lupus PBMCs *in vitro* reduced the expression of IFN inducible genes (48) and delivery to lupus-prone BXSB mice *in vivo* reduced the production of pro-inflammatory cytokines and autoantibodies (49). Thus miR-146a reduction is a biomarker for disease activity and a potential therapeutic target. Another interesting observation in the NZB/W model is the IFN induced expression of miR-15a in the spleens of treated mice; this is associated with downregulation of PAX5 and the emergence of autoantibodies and plasma cells. Since PAX5 is a negative regulator of miR-15a, the upregulation of miR-15a may be an early biomarker for IFN induction of plasma cells (35).

## Using the IFNα Accelerated Lupus Model to Test Therapeutics for SLE Nephritis

### IFNα kinoid

IFNα kinoid is an IFNα derived immunogen that triggers a strong but transient production of neutralizing antibody against IFNα (50). In a proof of principle experiment, prophylactic administration of kinoid delayed IFNα induced immune complex formation, proteinuria, and death in NZB/W F1 mice (50). It is worth noting that not all kinoid immunized mice mounted a substantial humoral response to IFNα and only the ones with antibody levels above a certain threshold showed delayed clinical manifestations. Moreover, a sustainable protective effect required prolonged production of anti-IFNα antibody, suggesting that periodic booster injections might be required to achieve a long-term antibody dependent clinical benefit. The success of this study led to the development of a human IFNα kinoid that induces antibodies neutralizing all 13 subtypes of human IFNαs (51). This kinoid has been shown to reduce the IFNα signature in lupus patients (52).

### Biologic therapies

TACI-Ig is a fusion protein that inhibits the BAFF/APRIL signaling pathway. The treatment of pre-autoimmune NZB/W F1 mice with TACI-Ig significantly delayed proteinuria onset and substantially prolonged the survival of the mice (53). TACI-Ig treatment achieved a similar clinical outcome in IFNα induced NZB/W F1 mice, although the survival benefit was only apparent when the treatment was started concomitant with IFN administration and was no longer effective if it was delayed until autoantibodies emerged (54). TACI-Ig treatment did not affect germinal center formation, autoantibody production, renal deposition of immune complexes, or pro-inflammatory cytokine expression in lymphoid organs however it was associated with a decrease in renal inflammation, prevention of activation of resident renal macrophages, and a decrease in renal and serum levels of TNF (54).

CTLA4-Ig, a drug that interrupts CD28-B7 interactions, prevents disease onset in NZB/W F1 mice (55, 56). In contrast, CTLA4-Ig at standard-dose failed to prevent or delay the onset of nephritis in Ad-IFNα treated mice despite preventing T and B cell activation, GC formation, and the production of pathogenic IgG2a anti-dsDNA antibodies (54). Resistance to standard-dose CTLA4-Ig was associated with the persistence of pathogenic IgG3 autoantibodies that were attenuated only after administration of high-dose CTLA4-Ig. Although the mice treated with high-dose CTLA4-Ig eventually died of nephritis, this treatment markedly delayed proteinuria onset and protected the mice from interstitial inflammation.

Ad-IFNα treatment in NZB/W F1 mice results in elevated renal expression and increased serum levels of TNFα concomitant with the onset of nephritis, making these mice an ideal model to study the efficacy of TNF receptor 2 (TNFR2)-Ig (57). TNFR2-Ig treatment delayed the onset of nephritis and prolonged survival of IFN accelerated mice without affecting autoantibody production or systemic immune activation. Similar to the observations with TACI-Ig, the therapeutic effect of TNFR2-Ig was achieved through inhibiting the renal response to immune complex deposition. The upregulation of a panel of chemokines in response to renal immune complex deposition was blocked by TNFR2-Ig treatment, resulting in diminished recruitment of periglomerular and interstitial F4/80^hi^ macrophages. In addition, renal endothelial cell activation and oxidative stress were decreased in the mice treated with TNFR2-Ig.

CD137 (4-1BB) is an inducible T cell costimulatory receptor belonging to the TNF receptor superfamily. It is expressed on activated CD4 and CD8 T cells, and promotes the proliferation of these cells. Treatment of NZB/W F1 mice with an agonistic anti-CD137 antibody significantly delays the onset of nephritis and prolongs survival (58). The therapeutic benefit is associated with inhibition of IgG but not IgM anti-dsDNA antibodies (58), consistent with an effect of anti-CD137 on T cell dependent but not T cell independent humoral responses (59). In preliminary experiments, we have shown that anti-CD137 antibody treatment of IFN induced NZB/W mice markedly delays the formation of germinal centers and the development of IgG2a and IgG3, but not IgM anti-dsDNA antibodies and greatly protects the kidneys from glomerular and interstitial injury (unpublished data), similar to the observations in NZB/W F1 mice that develop disease spontaneously. As with TACI-Ig, maximal benefit was achieved if the anti-CD137 treatment was started within 1 week of administration of Ad-IFNα.

### Remission induction therapies

Triple therapy with cyclophosphamide (CTX), anti-CD40L, and CTLA4-Ig induces remission in a high percentage of NZB/W F1 mice with established nephritis (60). A similar percentage of Ad-IFNα treated mice entered remission after this therapy but they relapsed rapidly (54). Mice treated with high-dose Ad-IFNα relapsed faster than mice treated with low dose Ad-IFNα and the latter relapsed faster than conventional NZB/W F1 mice (54, 60). Production of anti-dsDNA antibodies and glomerular deposition of IgG immune complex in Ad-IFNα treated NZB/W F1 mice were markedly reduced (54) by triple therapy, consistent with the observation that IFNα induces predominantly short-lived plasma cells which are susceptible to cytotoxic reagents (34). Nevertheless, in the high-dose IFN group, new autoreactive plasma cells formed as soon as the triple therapy drugs dissipated from the serum and this was associated with reaccumulation of renal immune complexes and rapid disease relapse. This is in contrast to conventional NZB/W F1 mice whose renal deposition of immune complexes was not reversed by the triple therapy (60) but whose renal response to immune complex deposition was markedly attenuated.

## The Utility of the IFNα Accelerated Lupus Model

Studies of therapeutic interventions in many strains of lupus-prone mice, such as NZB/W mice, are hampered by the stochastic disease onset and the length of time needed for the mice to develop spontaneous disease. In contrast, IFNα induced disease has a relatively synchronized onset and highly reproducible disease progression, allowing the study of therapies on defined stages of disease including remission induction studies. In addition, the IFNα induced model requires less time to develop clinical manifestations and has a shorter window from disease onset to death, allowing the therapies to be tested in a compact time frame.

At the same time, one needs to be aware that Ad-IFNα treated NZB/W F1 mice are not merely a hastened version of the spontaneous lupus model but they possess some distinct features. For instance, Ad-IFNα treated NZB/W F1 mice almost completely lack the long-lived plasma cells that exist in abundance in the spleen and bone marrow of the conventional NZB/W F1 mice (34) and therefore may not be a suitable model to test therapies targeting these pathogenic cells. Similarly, the modest renal infiltration with inflammatory cells renders the IFNα model less attractive for studies focused on inhibitors of leukocyte trafficking. Finally Ad-IFNα treatment also alters the response of disease to therapies that are highly effective in the spontaneous disease. Such therapies are for the most part only effective in the IFN model if they are administered prophylactically. This may reflect a more dynamic inflammatory environment in IFNα induced disease, rendering the disease more resistant to therapeutic intervention. Whether IFN signature^hi^ patients are similarly more resistant to drug interventions than IFN signature^lo^ patients needs to be determined in the context of clinical trials. It is of interest in this regard that preliminary studies of one anti-IFN agent showed no effect on autoantibodies and demonstrated efficacy only in the IFN signature^lo^ patient group (61, 63). By contrast, preliminary data from a second trial of a different anti-IFN agent showed a trend towards a better outcome in the patients with a high IFN signature (64). Further clinical trials are in progress. Overall Ad-IFNα treated mice are a reliable but stringent model to test new therapies for lupus nephritis. The difference in their response to therapies may help to predict proper intervention for patients with an IFN signature.

## Conflict of Interest Statement

The authors declare that the research was conducted in the absence of any commercial or financial relationships that could be construed as a potential conflict of interest.
